# Crystallization Phase Regulation of BaO-CaO-SiO_2_ Glass-Ceramics with High Thermal Expansion Coefficient

**DOI:** 10.3390/ma18071403

**Published:** 2025-03-21

**Authors:** Haonan Hu, Yongyuan Liang, Qifan Guan, Feng Liu, Mingsheng Ma, Yongxiang Li, Zhifu Liu

**Affiliations:** 1 CAS Key Laboratory of Inorganic Functional Materials and Devices, Shanghai Institute of Ceramics, Chinese Academy of Sciences, Shanghai 201899, China; 2 Center of Materials Science and Optoelectronics Engineering, University of Chinese Academy of Sciences, Beijing 100049, China; 3 Zhejiang Siramic-Tech Co., Ltd., Dongyang 322118, China; 4 Institute of Materials Research, Tsinghua Shenzhen International Graduate School, Tsinghua University, Shenzhen 518055, China; 5 School of Engineering, RMIT University, Melbourne, VIC 3001, Australia

**Keywords:** glass-ceramics, crystallization, thermal expansion, dielectric loss, packaging substrate

## Abstract

In this work, the influence of Ba/Ca ratios on the BaO-CaO-SiO_2_ (BCS) glass network structure, crystallization phases, and coefficient of thermal expansion (CTE) was investigated. As the Ba/Ca ratio increases, the Q^n^ units in the glass network structure have undergone significant changes. The Q^4^ units in the BCS glass network transform into Q^3^ units, indicating the reduction of the glass network connectivity. The variation in the Ba/Ca ratio leads to a change in the crystallization phases of BCS glass-ceramics sintered at a temperature higher than Tc (crystallization temperature). The addition of α-SiO_2_ (quartz) could regulate the crystallization phases and their ratio of the barium silicates (BaSi_2_O_5_, Ba_2_Si_3_O_8_, and Ba_5_Si_8_O_21_) in the BCS glass-ceramics. An abundant orthorhombic BaSi_2_O_5_ phase can be obtained in the BCS glass-ceramics with 15 wt% α-SiO_2_ calcinated over 875 °C. The α-SiO_2_ modified BCS glass-ceramics exhibited excellent properties (CTE = 12.10 ppm/°C, ε_r_ = 7.49 @ 13.4 GHz, tanδ = 4.96 × 10⁻⁴, Q × *f* = 27,034 GHz) sintered at optimized conditions, making it a promising candidate material for RF module and electronic packaging substrate.

## 1. Introduction

Multilayered ceramic technology has been widely used in electronic packaging, radio frequency (RF) components, and integrated modules [1,2,3]. The key properties of ceramic materials for these applications include low sintering temperature, low dielectric constant (ε_r_), and low dielectric loss (tanδ) [4,5]. Low sintering temperature enables the material to cofire with the metal electrode (Cu/Au/Ag) for multilayer integration [6]. A low dielectric constant leads to a small propagation delay of electromagnetic signals. The low dielectric loss would reduce the decay of electromagnetic signals and the thermal effect in packaging [7]. Furthermore, when substrates or modules are mounted onto printed circuit boards (PCBs) through ball grid arrays (BGAs), the difference in the coefficient of thermal expansion (CTE) between ceramic materials and PCBs (CTE~12–20 ppm/°C) may result in potential solder joint fatigue [8,9]. On the other hand, the high-throughput wireless data communication of 5G/6G systems would induce more pronounced thermal effects in integrated modules. To enhance the reliability of integrated systems necessitates the use of multilayered ceramic substrates with a high CTE [10,11,12].

Strategies to enhance the CTE of ceramic materials for packaging or integrated modules primarily focus on adjusting the composition of glass-ceramics and the crystallized phase to regulate their thermal expansion behavior. Studies on various glass-ceramics systems such as BaO-Al_2_O_3_-B_2_O-SiO_2_ [13,14,15,16,17], CaO-BaO-Al_2_O_3_-B_2_O_3_-SiO_2_ [18], CaO-B_2_O_3_-SiO_2_ [19], and Li_2_O-Al_2_O_3_-SiO_2_ [20] have demonstrated that the incorporation of phases with higher CTE (e.g., barium silicate and calcium barium silicate; see Table 1) could effectively increase the CTE of these materials. However, many studies revealed a trade-off between CTE and dielectric loss because of the complicated crystallization process, uncontrollable crystallization phase, and microstructure of glass-ceramic [13,14,17]. Previous works suggest that network-modifying ions (e.g., Ca^2+^, Sr^2+^, Ba^2+^, Mg^2+^) doping in glass can adjust the network connectivity and introduce the mixed alkaline earth effect to regulate both thermal expansion and dielectric properties [14,16,21,22,23,24]. Fillers and some additives could also modify the crystallization kinetic process and tune the crystallization phase of glass-ceramics [17]. It would be favorable for obtaining glass-ceramic with both high CTE and low dielectric loss if a large amount of high CTE low loss phase can be achieved by composition and thermal process control.

The BaO-CaO-SiO_2_ (BCS) glass-ceramics system could precipitate high thermal expansion coefficient phases, such as Ba_5_Si_8_O_21_, Ba_3_Si_5_O_13_, Ba_2_Si_3_O_8_, and BaSi_2_O_5_ [25,26,27]. Ghosh et al. [28] reported that the thermal expansion coefficient of the BCS glass-ceramics system can reach 9.5–13.0 ppm/°C by adjusting the composition to modify crystallization. Li et al. [29] found that the addition of Al_2_O_3_ and B_2_O_3_ to the BCS glass-ceramics system can achieve low dielectric loss (tanδ < 0.001). However, the addition of Al_2_O_3_ and B₂O₃ suppresses the precipitation of BaSi_2_O_5_, and the α-cristobalite/β-cristobalite phase transition leads to nonlinear thermal expansion, which can also cause thermal mismatch in electronic packaging substrates. The BaSi_2_O_5_ phase has garnered considerable attention due to its high CTE and low dielectric loss. Therefore, achieving a high concentration of the BaSi_2_O_5_ crystallization phase in the BCS glass-ceramics system is key to realizing both high thermal expansion and low dielectric loss.

**Table 1:** CTE and dielectric properties of high thermal expansion calcium silicate and barium silicate Phases.

Compounds	CTE (ppm/°C)	Q × *f* (GHz)	ε_r_	Reference
CaSiO_3_	11.2	29,300	6.9	[30,31]
Ca_2_SiO_4_	8.5	26,100	8.6	[30,31]
BaSiO_3_	12.5	6600	11.1	[32,33]
Ba_2_Si_3_O_8_	11.7	29,800	8.2	[32]
Ba_5_Si_8_O_21_	10.6	16,700	7.3	[32]
Ba_3_Si_5_O_13_	11.8	12,500	6.9	[32]
BaSi_2_O_5_	14.0	59,500	6.7	[32,33]

In this work, the BaO-CaO-SiO_2_ glass-ceramics with different Ba/Ca ratios were investigated. The effects of Ba/Ca ratios on glass microstructure, crystallization, and CTE of BCS glass-ceramics were systematically investigated. α-SiO_2_ was incorporated into BCS glass-ceramics to regulate the phase compositions and dielectric properties using a solid-state process. An abundant orthorhombic BaSi_2_O_5_ phase can be obtained in the BCS glass-ceramics modified by α-SiO_2_. Excellent performance was obtained from the modified BCS glass-ceramics.

## 2. Materials and Methods

BCS glasses with compositions of xBaO-yCaO-45SiO_2_ (mol%, where x + y = 55, and x/y ratios are 30/25, 35/20, 40/15, 45/10, and 50/5) were synthesized using a traditional melt-quenching method [26]. Reagent-grade BaCO_3_ (99%), CaCO_3_ (99%), SiO_2_ (99%), and α-SiO_2_ (99.9%) powders (China National Pharmaceutical Group Corporation, Beijing, China) were used as raw materials. The resulting glass fragments were ball-milled in ethanol for 24 h. The BCS glass powders with 5 wt%, 10 wt%, 15 wt%, and 20 wt% α-SiO_2_ addition were prepared. The BCS glass-ceramics powders with 15 wt% α-SiO_2_ addition, respectively, were prepared by calcining the mixed powders at 875 °C for 2 h and then subjected to a second round of ball-milling for 24 h. Polyvinyl alcohol (PVA) was added to the glass or composites to prepare the sintered bulk samples, and then, the granulated mixture was uniaxially pressed at 50 MPa into disks (13 mm in diameter) and rods (5 mm × 5 mm × 30 mm). After the removal of the PVA binder by heating at 450 °C for 2 h, the disks and rods were sintered at temperatures ranging from 700 °C to 1000 °C for 2 h, with a heating rate of 2 °C/min.

The volume density of the sintered glass-ceramics was determined using the Archimedes displacement method. Differential scanning calorimetry (DSC, NETZSCH 404, Selb, Germany) was employed to analyze the endothermic and exothermic peaks of the glass. The crystallization phases were characterized by X-ray diffraction (XRD, BRUKER D8 ADVANCE, Ettlingen, Germany). The microstructure and elemental distribution of the sintered samples were observed using a scanning electron microscope equipped with energy disperse spectroscopy (SEM&EDS, Verios G4, Waltham, MA, USA). Raman spectroscopy (InVia, Renishaw, Gloucestershire, UK) was utilized to analyze the glass microstructure. The network structure of the glass was analyzed using magic angle spinning nuclear magnetic resonance (MAS-NMR, BRUKER AVANCE IIIHD 500M, Ettlingen, Germany). The coefficient of thermal expansion (CTE) of the samples was measured using a thermomechanical analyzer (NETZSCH 402 F3, Selb, Germany). The dielectric constant (ε_r_) and low dielectric loss tangent (tanδ) of sintered ceramics were measured at room temperature using the Hakki–Coleman method by a PNA Series Network Analyzer (AGILENT E8363A, Santa Clara, CA, USA). The Q × *f* value can be calculated using the Equation (1):

(1) $$Q\times f=\frac{f}{\tan\delta }$$

where Q is the quality factor, *f* is the resonant frequency, and tanδ is the dielectric loss tangent.

## 3. Results and Discussion

### 3.1. Effect of Ba/Ca Ratio on Structure and Crystallization of BCS Glasses

**Figure 1:**
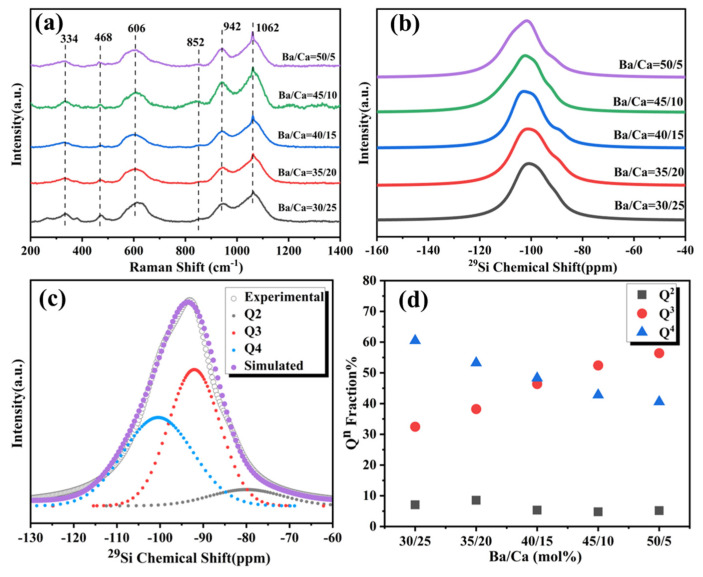
Structural analysis of BCS glasses. (**a**) Raman spectra of as-prepared BCS glasses. (**b**) ^2^⁹Si MAS NMR spectra of as-prepared BCS glasses. (**c**) Gaussian fitting of the ^2^⁹Si MAS NMR spectra peaks for Q^n^ analysis. (**d**) Calculated Q^n^ group proportions in the glass structures with different Ba/Ca ratios.

The Raman spectra of BCS glasses with different Ba/Ca ratios (Figure 1a) primarily exhibit three main bands at 300–500 cm⁻^1^, 550–650 cm⁻^1^, and 850–1200 cm⁻^1^. The bands with peaks at 334 cm⁻^1^ and 468 cm⁻^1^ are attributed to the [Si–O] rocking and bending vibrations [34]. The band peaked at 606 cm⁻^1^ is ascribed to the [Si–O–Si] bending vibration in depolymerized structural units [35], and the band at 850–1200 cm⁻^1^ originates from the [Si–O–Si] bending vibration of 3- or 4-membered silica rings in the glass [36,37]. As shown in Figure 1a, the Ba/Ca ratio in the BCS glass composition has minimal effect on the low-frequency bands. The variation is observed in the peak intensity within the 900–1200 cm⁻^1^ range. Specifically, the enhanced peak at around 1062 cm^−1^ indicates that changes in the Ba/Ca ratio affect the connectivity of the glass network, which is closely associated with the Q^n^ units [38].

The local environment of the Si element was examined using MAS-NMR spectra to further confirm the effect of the Ba/Ca ratio on the connectivity of the glass network. The ^29^Si NMR spectra (Figure 1b) of BCS glasses all exhibit a broad peak in the range of −120 ppm to −70 ppm, indicating that there is a distribution of Q^n^ units (where Q^n^ refers to the [SiO_4_] tetrahedra with n bridging oxygens, n = 0, 1, 2, 3, 4). This broad chemical shift peak observed in the ^2^⁹Si NMR spectra (−120 ppm to −70 ppm) can be Gaussian-fitted into three peaks (Figure 1c). The Q^0^ and Q^1^ units are primarily located at −66 ppm to −62 ppm and −76 ppm to −68 ppm with very low detected intensities [37]. In contrast, the Q^2^, Q^3^, and Q^4^ units are distributed across the spectra: the peak at −110 ppm to −95 ppm corresponds to the Q^4^ unit in the glass network, the peak at −95 ppm to −85 ppm corresponds to the Q^3^ unit, and the peak at −85 ppm to −75 ppm corresponds to the Q^2^ unit [39,40]. The proportion of Q^n^ units can be determined by calculating the area of the Gaussian-fitted peaks (Figure 1d). As the Ba/Ca ratio increases, the content of Q^2^ units remains nearly unchanged. While, the content of Q^3^ units increases from 32% to 56%, and the content of Q^4^ units decreases from 60% to 40%. This demonstrates that an increase in the Ba/Ca ratio induces the conversion of Q^4^ units to Q^3^ units in the BCS glass network, leading to a decrease in the number of bridging oxygens. It has been reported that different Q^n^ units form various anionic structures [35,41,42]. Different anionic structures would result in different nucleation, crystallization phenomena, and crystallization phases after heat treatment.

**Figure 2:**
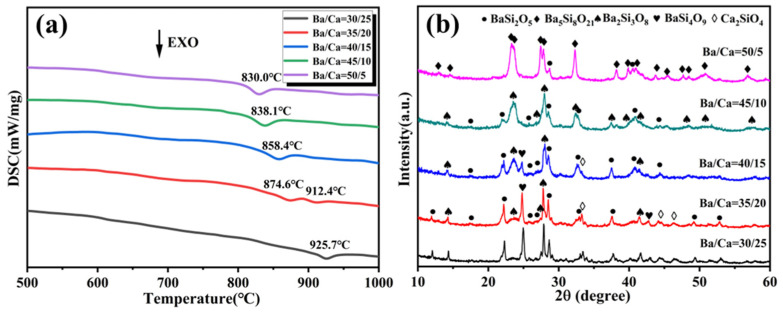
(**a**) Differential scanning calorimetry (DSC) curves of BCS glasses. (**b**) XRD patterns of BCS glass-ceramics sintered above the crystallization temperatures.

The effect of the Ba/Ca ratio on the thermal behavior of BCS glass was further investigated using differential scanning calorimetry (DSC). Figure 2a shows the DSC curves of glass powders with different Ba/Ca ratios, measured at a heating rate of 10 K/min. The DSC curves of various BCS glass compositions exhibit one or two distinct exothermic peaks, which correspond to the crystallization peaks of glasses with different compositions. As the Ba/Ca ratio increases, the crystallization temperature of the BCS glass decreases from 925.7 °C to 830 °C. This is attributed to the reduction in the connectivity of the glass network, which releases more “free oxygen”, disrupting the Si-O bonding network and relaxing the glass structure [15]. The Ba^2^⁺ and Ca^2^⁺ ions have a depolarizing effect on the Si-O bonds in the network, weakening the strength of the Si-O bonds. Ba^2^⁺ has a stronger weakening effect than Ca^2^⁺ [12,43]. According to the relationship between the sintering temperature and the volume density of BCS glass-ceramics (Figure S1), BCS glasses with different Ba/Ca ratios can all achieve their maximum bulk density at a sintering temperature of no more than 775 °C. Since the DSC curves show that the crystallization temperatures of all BCS glass compositions are above 775 °C, the sintering process was performed above the crystallization temperature of all samples to obtain BCS glass-ceramics.

The crystallization temperatures of different glass compositions are determined by the temperature of the first crystallization peak in their DSC curves. The crystallization temperatures for the Ba/Ca ratios of 30/25, 35/20, 40/15, 45/10, and 50/5 are 925 °C, 875 °C, 875 °C, 850 °C, and 850 °C, respectively. Figure 2b shows the main crystallization phase changes of BCS glass-ceramics with different Ba/Ca ratios at their crystallization temperatures. Overall, the main crystalline phases formed in the glass of each composition include BaSi_2_O_5_ (JCPDS#72-0171), Ba_5_Si_8_O_21_ (JCPDS#35-0766), Ba_2_Si_3_O_8_ (JCPDS#27-1035), BaSi_4_O_9_ (JCPDS#83-0958), and Ca_2_SiO_4_ (JCPDS#36-0642). In BCS glasses with Ba/Ca ratios of 30/25, 35/20, and 40/15, the main crystallization phases include BaSi_2_O_5_, Ba_2_Si_3_O_8_, and BaSi_4_O_9_, along with the formation of minor amounts of Ca_2_SiO_4_ phases. As the Ba/Ca ratio increases, Ca_2_SiO_4_ phases gradually disappear, and the barium silicate phases in the BCS glass also undergo transformations. At a Ba/Ca ratio of 45/10, the primary crystallization phases are BaSi_2_O_5_ and Ba_2_Si_3_O_8_. At a Ba/Ca ratio of 50/5, monoclinic Ba_5_Si_8_O_21_ becomes the dominant crystallization phase.

The phases precipitated in glass are closely related to the glass network structure. It has been reported that all lattice structures of barium silicates contain [SiO_4_] tetrahedra. Both Ba_2_Si_3_O_8_ and Ba_5_Si_8_O_21_ have tetrahedral chains or one-dimensional structures, while BaSi_2_O_5_ has layered or two-dimensional structures [44]. For both one-dimensional and two-dimensional structures, the tetrahedral chains are *zwieier*, with two [SiO_4_] tetrahedra in the repeating unit [45]. The structure of barium silicate can be described as Ba_1+1/M_ [Si_2_O_5+1/M_]. In the case of BaSi_2_O_5_, its layered structure is composed of an infinite number of single chains (M = ∞) [32]. This structure is similar to the short-range or medium-range ordered structure composed of Q^4^ units [1]. Therefore, when Q^4^ units dominate the glass network structure, BaSi_2_O_5_ preferentially nucleates and precipitates. As the Ba/Ca ratio increases, Q^3^ units replace Q^4^ units, and the corresponding crystalline phase Ba_5_Si_8_O_21_ becomes the preferred nucleation phase. This is also attributed to the structural similarity between the Ba_5_Si_8_O_21_ phase and the glass composition. The CTE value of these types of barium silicates is closely related to the complexity of the crystal structure, phase composition, and the tilting and distortion of [SiO_4_] tetrahedra [32].

**Figure 3:**
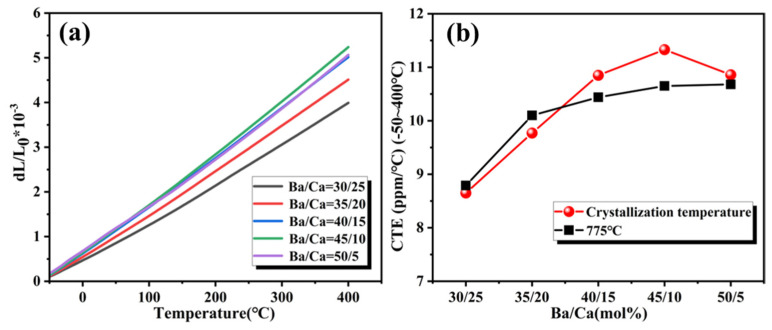
(**a**) Thermal expansion curves of BCS glass-ceramics sintered at crystallization temperature. (**b**) CTE of BCS glasses with different Ba/Ca ratios sintered at different temperatures in the temperature range of −50 °C to 400 °C.

The thermal expansion property was investigated to clarify the effect of the Ba/Ca ratio and crystallization phase composition on the properties. The thermal expansion of BCS glasses with different Ba/Ca ratios and sintered at different conditions are illustrated in Figure 3. The thermal expansion curves (Figure 3a) for all compositions demonstrate good linearity. After sintered at 775 °C, the CTE of sintered BCS glass increases with the increase of Ba/Ca ratio, rising from 8.8 ppm/°C to 10.7 ppm/°C at the range of −50 °C to 400 °C. This increase is primarily attributed to the change in the chemical bond strength and reduced network connectivity in the BCS glass as the Ba/Ca ratio increased [46]. Alkali earth meta ions exert effects on the Si-O bonds within the network and increase the availability of “free oxygen” to break the Si-O network and relax the glass structure, thereby weakening the strength of the Si-O bonds [44,45]. Notably, Ba^2^⁺ has a stronger weakening effect on Si-O bonds than Ca^2^⁺ due to its larger ionic radius and lower field strength compared to Ca^2^⁺. The increase in the Ba/Ca ratio would also result in an increased concentration of non-bridging oxygen (NBO). Raman spectroscopy and solid-state NMR analyses confirm that this alteration is closely linked to the Q^n^ units. The increase in the Ba/Ca ratio induces the transition from Q^4^ units to Q^3^ units, thereby reducing the connectivity of the glass network. Lower network connectivity in glass results in a higher thermal expansion coefficient [46].

Upon increasing the sintering temperature to above the crystallization temperature, the precipitation of the low-expansion phase Ca_2_SiO_4_ (CTE = 8.5 ppm/°C) results in a decrease in CTE of the BCS glass-ceramics for these with low Ba/Ca ratios. Obviously, the CTE of the glass-ceramic is higher than its glass counterpart for those with Ba/Ca ratios higher than 40/15. The highest CTE value, 11.3 ppm/°C (−50 to 400 °C), is observed from glass-ceramic with a Ba/Ca ratio of 45/10. The CTE of BCS glass-ceramics exhibits a nonlinear variation. This is primarily attributed to the changes in the crystallization phases. As the Ba/Ca ratio increases, the low-expansion phase Ca_2_SiO_4_ (CTE = 8.5 ppm/°C) gradually disappears, which contributes to the increase in the CTE of the BCS glass-ceramics. Concurrently, as the Ba/Ca ratio continues to rise, the crystalline phases of barium silicate (BaSi_2_O_5_, Ba_5_Si_8_O_21_, Ba_2_Si_3_O_8_, BaSi_4_O_9_) undergo structural changes. At a Ba/Ca ratio of 45/10, the primary crystallization phases are BaSi_2_O_5_ and Ba_2_Si_3_O_8_. At a Ba/Ca ratio of 50/5, monoclinic Ba_5_Si_8_O_21_ becomes the dominant crystallization phase. Since the CTE of Ba_5_Si_8_O_21_ (10.6 ppm/°C) is lower than that of BaSi_2_O_5_ (14.0 ppm/°C) and Ba_2_Si_3_O_8_ (11.7 ppm/°C), this results in a reduction in the CTE of BCS glass-ceramics with a Ba/Ca ratio of 50/5.

### 3.2. Phase Regulation of BCS Glass-Ceramics by α-SiO_2_

The phase composition of glass-ceramics plays a crucial role in determining their microwave dielectric and thermal properties. To develop material with both a high thermal expansion coefficient and low dielectric loss, we added α-SiO_2_ (quartz) to BCS glass (Ba/Ca ratio is 45/10) to tune the sintering temperature and properties. Interestingly, it was found that a substantial amount of orthorhombic BaSi_2_O_5_ (CTE = 14.0 ppm/°C, Q × *f* = 59,500 GHz) precipitates after sintering at 875 °C when the α-SiO_2_ content exceeds 15 wt% (Figure S2), which is beneficial for obtaining high thermal expansion and low dielectric loss. So, more detailed work was carried out to check the phase regulation effect of α-SiO_2_ on the BCS glass-ceramics.

**Figure 4:**
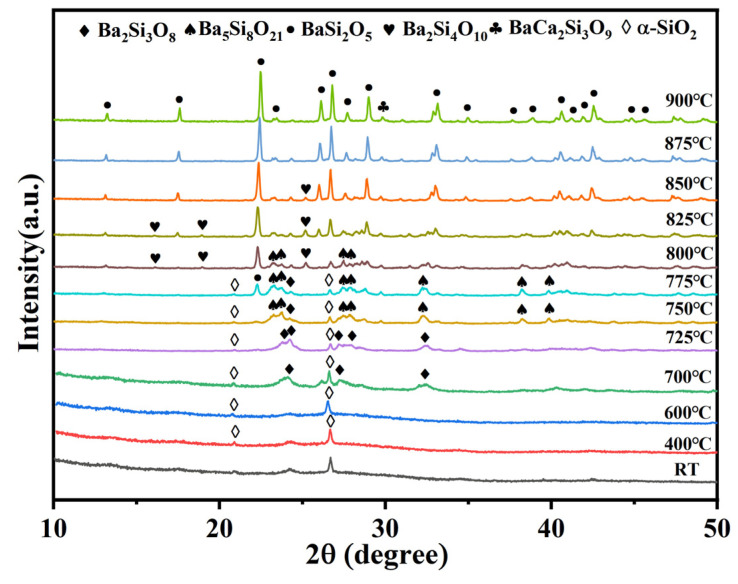
XRD patterns of α-SiO_2_ modified BCS glass sintered at 400–900 °C.

XRD was used to confirm the crystallization phases of the 15 wt% α-SiO_2_ modified BCS glass powders after sintered at temperatures ranging from 400 °C to 900 °C (Figure 4). The BCS glass did not exhibit any phase transitions below 600 °C. After reaching 700 °C, Ba_2_Si_3_O_8_ began to precipitate, and the diffraction peaks of α-SiO_2_ gradually diminished. Ba_5_Si_8_O_21_ began to form after 750 °C, and at 775 °C, Ba_5_Si_8_O_21_ reacted with SiO_2_ to form BaSi_2_O_5_. This suggests that the barium silicate phases undergo transformations as outlined in Equations (2)–(4) by reacting with SiO_2_. Above 800 °C, a small amount of Ba_2_Si_4_O_10_ (a high-temperature form of BaSi_2_O_5_) begins to form. This is attributed to the higher tetrahedral orientation diversity in the structure of Ba_2_Si_4_O_10_ compared to orthorhombic BaSi_2_O_5_, which results in a lower energy barrier for the precipitation of Ba_2_Si_4_O_10_ [47,48]. However, as the temperature increases, Ba_2_Si_4_O_10_ gradually transforms into orthorhombic BaSi_2_O_5_. When the sintering temperature exceeds 875 °C, the phase composition of the BCS glass-ceramics does not change anymore. The composites predominantly consisted of BaSi_2_O_5_ with a minor amount of BaCa_2_Si_3_O_9_.

(2) $$T<750\;{}^{\circ}C:\;Ba_{2}Si_{3}O_{8}+SiO_{2}$$

(3) $$T\geq 750\;{}^{\circ}C:\;2.5Ba_{2}Si_{3}O_{8}+0.5SiO_{2}=Ba_{5}Si_{8}O_{21}$$

(4) $$T\geq 775\;{}^{\circ}C\text{:}\;Ba_{5}Si_{8}O_{21}+2SiO_{2}=5BaSi_{2}O_{5}$$

**Figure 5:**
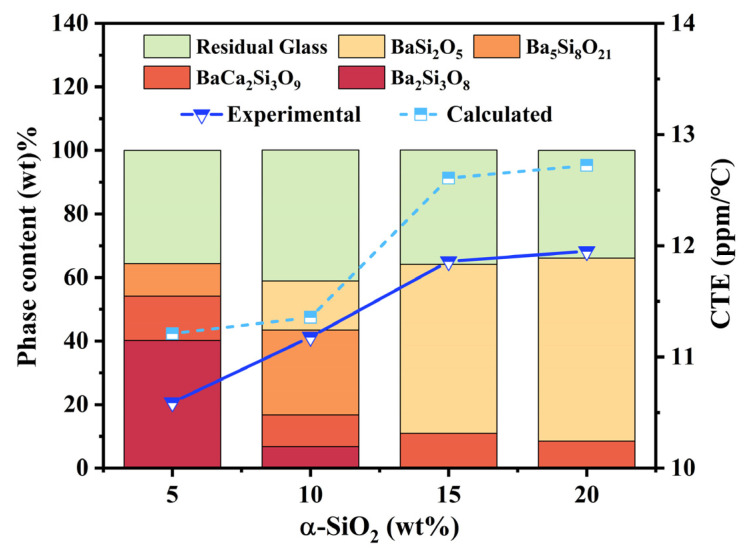
Comparison between calculated and experimental values of the coefficient of thermal expansion (CTE) for BCS glass-ceramics with different α-SiO_2_ additions.

The types and concentrations of crystalline phases in glass-ceramics are key factors in determining their coefficient of thermal expansion (CTE). The phase composition of the BCS glass-ceramics after the addition of α-SiO_2_ was further evaluated using Rietveld refinement analysis (Figure S3). As shown in Figure 5, the residual glass phase content in the BCS glass-ceramics is similar across all samples (ranging from approximately 34 wt% to 41 wt%). Additionally, when the α-SiO_2_ content exceeds 15 wt%, a substantial amount of orthorhombic BaSi_2_O_5_ (over 50 wt%) precipitates. Considering the effects of all phases in the glass-ceramics, the CTE can be calculated using the Equation (5) [49]:

(5) $$\alpha =\alpha_{g}w_{g}+\alpha_{1}w_{1}+\alpha_{2}w_{2}+\cdots +\alpha_{n}w_{n}$$

where *α* is the coefficient of thermal expansion (CTE) of the glass-ceramics, *α_g_*, *α*_1_, *α*_2_, …, *α_n_* are the CTEs of the glass phase and crystalline phase in the composition, *ω_g_*, *ω*_1_, *ω*_2_, …, *ω_n_* are the weight fractions of the glass phase and crystalline phase in the composition. The CTEs of the crystalline phases are listed in Table 1, and the CTE of the residual glass phase can be considered as the BCS glass (with a Ba/Ca ratio of 45/10) before crystallization (CTE = 10.65 ppm/°C; see Figure 3b). The comparison between the calculated and experimental CTE values is shown in Figure 5, which exhibits similar trends. The CTE of the BCS glass-ceramics increases with the addition of α-SiO_2_. This is attributed to the precipitation of the crystalline phase BaSi_2_O_5_, which has a higher CTE. As the content of α-SiO_2_ increases, the content of the BaSi_2_O_5_ crystalline phase also increases. In addition, there are discrepancies between the calculated CTE values and the experimental values. These discrepancies may arise from the presence of pores and defects within the glass-ceramics. The addition of α-SiO_2_ makes it more difficult for the BCS glass-ceramics to achieve dense sintering. The higher the addition of α-SiO_2_, the more pores are present in the BCS glass-ceramics (Figure S4).

### 3.3. Properties of the Modified BCS Glass-Ceramics

**Figure 6:**
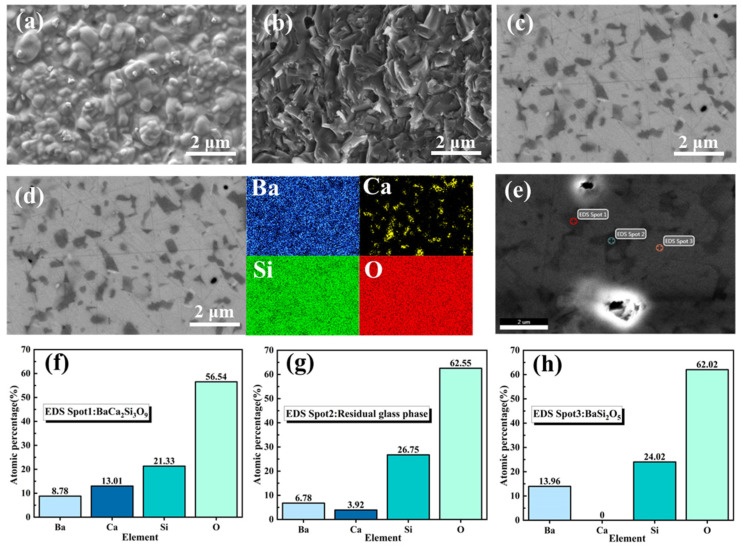
(**a**–**c**) SEM images of the α-SiO_2_ modified BCS glass-ceramics sintered at 1000 °C: (**a**) natural surface, (**b**) fracture surface, and (**c**) polished surface. (**d**–**h**) EDS analysis: surface scan (**d**) and point scan (**e**–**h**) results.

The sintering process was further optimized to obtain BCS glass-ceramics with superior properties and high density (selecting the BCS glass-ceramics composition with 15wt% α-SiO_2_ addition). The SEM images presented in Figure 6 illustrate the microstructure and elemental composition distribution of the α-SiO_2_ modified BCS glass-ceramics sintered at 1000 °C. As shown in Figure 6a, crystallized ceramic grains have precipitated, encircled by the residual BCS glass phase. On the polished surface (Figure 6c), three distinct phases with varying contrasts are discernible. The elemental composition and distribution of these phases were further investigated using energy-dispersive X-ray spectroscopy (EDS) (Figure 6d–h). Point-scan EDS analysis reveals that the three phases correspond to BaSi_2_O_5_, BaCa_2_Si_3_O_9_, and the residual glass phase, which is consistent with the XRD results. Additionally, the EDS surface scan demonstrates an enrichment of calcium in certain regions. While most crystallized areas exhibit a lack of calcium, further substantiating the significant precipitation of BaSi_2_O_5_.

**Figure 7:**
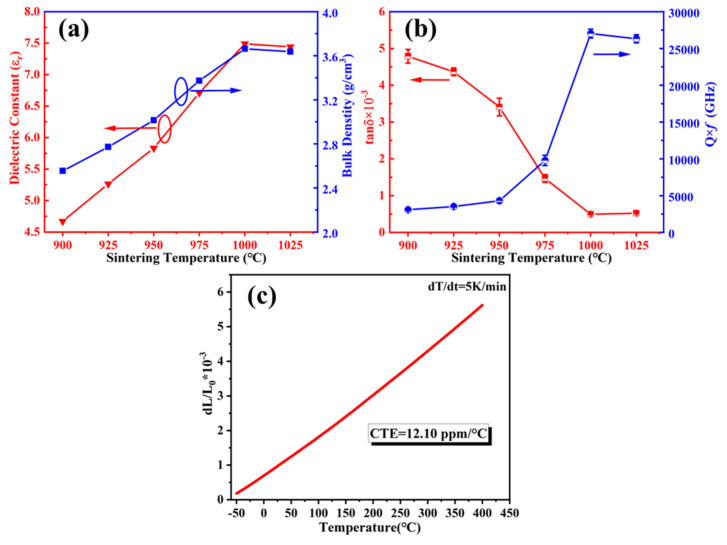
Properties of the α-SiO_2_ modified BCS glass-ceramics: (**a**) dielectric constant (ε_r_), (**b**) dielectric loss (tanδ), and (**c**) CTE.

The dielectric constant and loss of the sintered modified BCS glass-ceramics were measured and shown in Figure 7. As the sintering temperature increases, the dielectric constant (ε_r_) initially increases and then slightly decreases. This trend can be attributed to the reduction in porosity of the glass-ceramics composites. The highest dielectric constant (ε_r_ = 7.49) is observed at the sample with the maximum volume density (ρ = 3.66 g/cm^3^). Figure 7b shows the variation of the Q × *f* value and dielectric loss (tanδ) with sintering temperature. The dielectric loss decreases with increasing sintering temperature, reaching a minimum value at 1000 °C. The α-SiO_2_ modified BCS glass-ceramics exhibited the best dielectric properties (ε = 7.49 @ 13.4 GHz, tanδ = 4.96 × 10⁻^4^, Q × *f* = 27,034 GHz) sintered at 1000 °C. The precipitation of BaSi_2_O_5_ crystals and the reduction in the glass phase can significantly reduce the dielectric loss of the composites. The decrease of porosity and other possible defects with the increase of sintering temperature may also contribute to the low dielectric loss.

As shown in Figure 7c, the α-SiO_2_ modified BCS glass-ceramics exhibits a high coefficient of thermal expansion (CTE = 12.10 ppm/°C) after sintered at 1000 °C. This represents a notable increase compared to the CTE of the BCS glass-ceramics. The enhancement in CTE can be attributed to the incorporation of α-SiO_2_, which modifies the phase composition of the BCS glass-ceramics and promotes the precipitation of orthorhombic BaSi_2_O_5_. Compared with commercial ceramic packaging substrate materials (Table 2), the high CTE and excellent dielectric performance make the α-SiO_2_ modified BCS glass-ceramics a promising candidate for RF module and electronic packaging substrate applications.

## 4. Conclusions

In summary, the effect of the Ba/Ca ratios on the network structure of BCS glasses and their crystallization behavior was investigated. As the Ba/Ca ratio increases, the transformation from Q^4^ to Q^3^ units in the BCS glass network reduces the connectivity of the network, leading to an increase in CTE. The increase in the Ba/Ca ratio leads to a change in the crystallization phases of BCS glass-ceramics sintered at a temperature higher than Tc (crystallization temperature). The highest CTE was achieved from BCS glass-ceramics with Ba/Ca = 45/10. The crystallization phase composition of BCS glass-ceramics can be regulated by α-SiO_2_. Orthorhombic BaSi_2_O_5_ becomes the main phase treated at 875 °C with 15 wt% α-SiO_2_ addition. The α-SiO_2_ modified BCS glass-ceramics exhibited excellent properties (CTE = 12.10 ppm/°C, ε = 7.49 @ 13.4 GHz, tanδ = 4.96 × 10⁻⁴, Q × *f* = 27,034 GHz) sintered at optimized condition, demonstrating its potential for RF module and electronic packaging substrate applications.

## Figures and Tables

**Table 2:** Thermal expansion and dielectric properties of commercial ceramic packaging substrate materials and the material studied in this work.

Materials	CTE (ppm/°C)	tanδ × 10^−4^	ε_r_ @ 10 GHz	Reference
Dupont 951	5.8	15	7.5	[50]
Kyocera GL771	12.3	38	5.2	[50]
Kyocera GL773	11.7	25	5.8	[50]
Heraeus CT708	10.6	30	6.4	[6]
This work	12.1	4.96	7.5	

## Data Availability

The original contributions presented in this study are included in the article/Supplementary Materials. Further inquiries can be directed to the corresponding authors.

## Supplementary Materials

The following supporting information can be downloaded at https://www.mdpi.com/article/10.3390/ma18071403/s1, Figure S1. The bulk density of BCS glass-ceramics as functions of sintering temperature; Figure S2. XRD patterns of the BCS glass-ceramics with different α-SiO_2_ additions sintered at 875 °C; Figure S3. Rietveld refinement of BCS glass-ceramics with different α-SiO_2_ additions sintered at 875 °C. (a) 5 wt% α-SiO_2_. (b) 10 wt% α-SiO_2_. (c) 15 wt% α-SiO_2_. (d) 20 wt% α-SiO_2_; Figure S4. (a) SEM images of BCS glass-ceramics with different α-SiO_2_ additions sintered at 875 °C. (a) 5 wt% α-SiO_2_. (b) 10 wt% α-SiO_2_. (c) 15 wt% α-SiO_2_. (d) 20 wt% α-SiO_2_.
