# Supplementary material for: Crystallization Phase Regulation of BaO-CaO-SiO2 Glass-Ceramics with High Thermal Expansion Coefficient
Source: Materials (Basel). 2025 Mar 21;18(7):1403. doi: 10.3390/ma18071403 (PMC11989664; doi:10.3390/ma18071403)
Supplement: Supplementary file 1 [file materials-18-01403-s001.zip › materials-3509369-supplementary.pdf]

Crystallization Phase Regulation of BaO-CaO-SiO2 Glass-Ceramics with High Thermal Expansion Coefficient

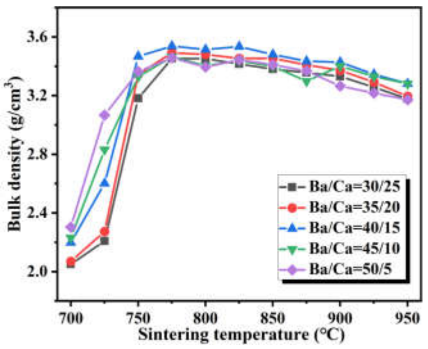

Figure S1. The bulk density of BCS glass-ceramics as functions of sintering temperature.

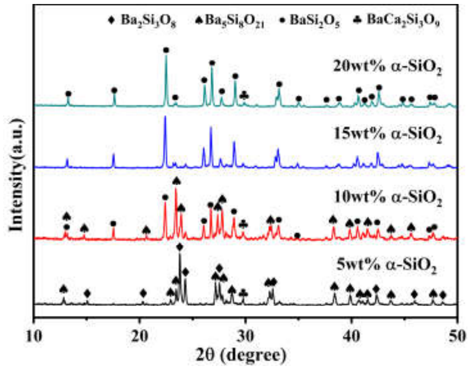

Figure S2. XRD patterns of the BCS glass-ceramics with different α-SiO_2 additions sintered at 875℃.

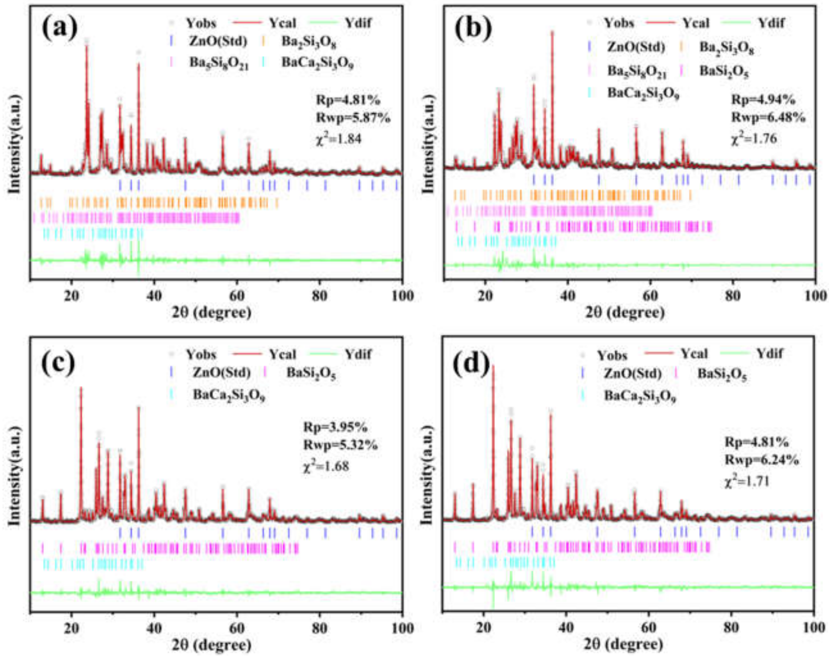

Figure S3. Rietveld refinement of BCS glass-ceramics with different α-SiO_2 additions sintered at 875°C. (a) 5 wt% α-SiO_2. (b) 10 wt% α-SiO_2. (c) 15 wt% α-SiO_2. (d) 20 wt% α-SiO_2.

The phase composition concentration of the BCS glass-ceramics after the addition of α-SiO2 was further evaluated using Rietveld refinement analysis. The weight fraction of the i-th component in a mixture of n phases was obtained using the equation (1):

$$W_i = \frac{S_i v_i Z_i M_i}{\sum (S_n v_n Z_n M_n)} \quad (1)$$

where W_i is the relative weight fraction of the *i*-th crystalline phase in the sample, S_i is the scale factor of the *i*-th phase, v_i is the unit cell volume, Z_i is the number of molecules in the unit cell, and M_i is the molecular weight of the *i*-th phase. Therefore, the relative weight fractions are determined after the scale factors and unit cell volumes are refined using the Rietveld program. Additionally, the residual glass phase content can be more accurately calculated by adding ZnO (16.7wt% of the total mass) using the external standard method. As calculated by equation (2):

$$X_g = \frac{100}{(100 - X_s)}(1 - \frac{X_s}{X_{sc}}) \quad (2)$$

where X_g is the relative weight fraction of the amorphous phase by using the external standard method, X_s is the relative weight fraction of the added external standard, X_{sc} is the relative weight fraction of the external standard obtained by Rietveld refinement analysis.

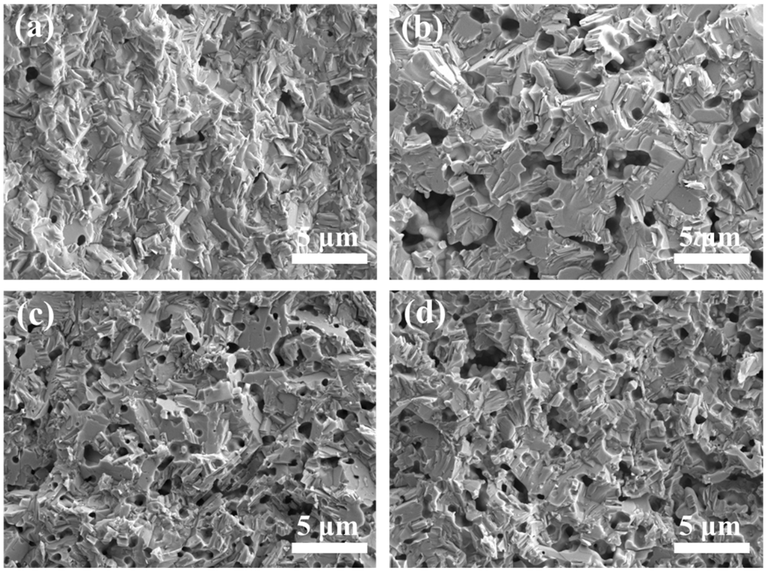

Figure S4. (a) SEM images of BCS glass-ceramics with different α-SiO_2 additions sintered at 875℃. (a) 5 wt% α-SiO_2. (b) 10 wt% α-SiO_2. (c) 15 wt% α-SiO_2. (d) 20 wt%α-SiO_2.
